# Hypnotic Effects of *Lactobacillus fermentum* PS150^TM^ on Pentobarbital-Induced Sleep in Mice

**DOI:** 10.3390/nu11102409

**Published:** 2019-10-09

**Authors:** Alexander Lin, Ching-Ting Shih, Chin-Lin Huang, Chien-Chen Wu, Ching-Ting Lin, Ying-Chieh Tsai

**Affiliations:** 1 Institute of Biochemistry and Molecular Biology, National Yang-Ming University, Taipei 11221, Taiwan; alexander.lin@cm-biopha.com; 2 Chung Mei Biopharma Co., Ltd., Taichung 40453, Taiwan; alexis.shih@cm-biopha.com; 3 Bened Biomedical Co., Ltd., Taipei 11221, Taiwan; hjenny@benedbiomed.com (C.-L.H.); allen@benedbiomed.com (C.-C.W.); 4 School of Chinese Medicine, China Medical University, Taichung 40402, Taiwan

**Keywords:** *Lactobacillus fermentum*, sleep, pentobarbital, caffeine, adenosine 1 receptor

## Abstract

The bidirectional communication between the gastrointestinal tract and the central nervous system appears to be functionally linked to the intestinal microbiome, namely the microbiome–gut–brain axis (MGBA). Probiotics with health benefits on psychiatric or neurological illnesses are generally called psychobiotics, and some of them may also be able to improve sleep by targeting the MGBA. This study aimed to investigate the effects of a psychobiotic strain, *Lactobacillus fermentum* PS150^TM^ (PS150^TM^), on sleep improvement by using a pentobarbital-induced sleep mouse model. Compared with the vehicle control group, the oral administration of PS150^TM^, but not the other *L. fermentum* strains, significantly decreased the sleep latency and increased the sleep duration of mice, suggesting strain-specific sleep-improving effects of PS150^TM^. Moreover, the ingestion of diphenhydramine, an antihistamine used to treat insomnia, as a drug control group, only increased the sleep duration of mice. We also found that the sleep-improving effects of PS150^TM^ are time- and dose-dependent. Furthermore, the oral administration of PS150^TM^ could attenuate a caffeine-induced sleep disturbance in mice, and PS150^TM^ appeared to increase the expression of the gene encoding the adenosine 1 receptor in the hypothalamus of mice, as assessed by quantitative real-time polymerase chain reaction. Taken together, our results present a potential application of PS150^TM^ as a dietary supplement for sleep improvement.

## 1. Introduction

Insomnia is a common disorder characterized by difficulty falling asleep and hardly maintaining sleep or waking up too early. Approximately 20–30% of adults have chronic insomnia problems (i.e., with a duration of at least 1 month), which is the most prevalent sleep disorder in the general population [1]. It is usually accompanied by psychiatric or physical diseases such as impaired attention, irritability, restlessness, anxiety, and stress or fatigue during wakefulness [2]. Insomnia also increases the risk of chronic diseases (e.g., hypertension, diabetes, stroke, and coronary artery diseases) as well as healthcare costs [3,4]. Although different drugs are used to treat insomnia, most of them are not recommended for long-term use because of their potential adverse reactions [3,5]. Thus, a search for alternative ways to treat insomnia is needed. Insomnia and gastrointestinal dysfunction have been reported to influence each other, implying a dynamic bidirectional relationship between sleep and gastrointestinal health [6]. Moreover, a circadian dysfunction is usually accompanied with intestinal dysbiosis and inflammatory responses, often making it is difficult to determine their causality [7,8].

Probiotics are defined as “live microorganisms which, when administered in adequate amounts, confer a health benefit on the host” [9]. Dietary-supplemented probiotics are well-known to help to avoid gastrointestinal infections, improve intestinal functions [10], alleviate allergies, and regulate immunity [11]. Besides of benefits to children and adults, probiotics supplementation during pregnancy and in the neonatal period might reduce some maternal and neonatal adverse outcomes [12]. In recent years, gut microbes have been shown to indirectly interact with the host’s central nervous system (CNS) through the gut–brain axis (GBA), which is a bidirectional communication system that integrates neural, hormonal, and immunological signaling between the gut and the brain [13,14]; thus, the concept of a microbiome–gut–brain axis (MGBA) was established [15]. Furthermore, certain probiotics identified as “psychobiotics” can modulate the MGBA to bring health benefits to hosts with psychiatric or neurological illnesses, which holds promise regarding the treatment of insomnia.

In this study, we attempted to identify probiotic strains with sleep-improving effects by using a pentobarbital-induced sleep mouse model. Numerous strains belonging to different *Lactobacillus* spp. were screened. Subsequently, a specific *Lactobacillus fermentum* strain, PS150^TM^, was found to have sleep-improving effects. The strain has previously been demonstrated to have psychotropic properties by altering the serotonergic pathway during stress conditions in rats [16]. Further experiments were performed to investigate the strain specificity and potential hypnotic effects of PS150^TM^ on pentobarbital-induced sleep mice.

## 2. Materials and Methods

### 2.1. Bacterial Strain, Media, and Growth Conditions

*L. fermentum* strains PS150^TM^, PCC (Sydney, Australia) [17], and ATCC 14931^T^ (the taxonomic type strain) [18] were inoculated in *Lactobacilli* Man Rogosa Sharpe (MRS; BD Difco), cultured at 37 °C for 18 h. For mouse experiments, the *L. fermentum* culture was harvested using centrifugation (10,000× *g*, 10 min), washed twice with sterile phosphate-buffered saline (PBS), and re-suspended with PBS to a final concentration of approximately 10^10^ colony-forming units (CFU)/mL.

### 2.2. Animals

Adult male C57BL/6J mice (6 weeks old) were purchased from the National Laboratory Animal Center (Taipei, Taiwan). First, the mice were accommodated in the specific pathogen-free room at the Laboratory Animal Center of National Yang-Ming University. The room was kept at 22 ± 1 °C, 55–65% humidity, and under a 12:12 h light-dark cycle. The mice were fed a commercial diet (5010 LabDiet) and sterile water ad libitum. The experiments were performed after 1 week of acclimation. All experiments were conducted following relevant guidelines and regulations and were pre-approved by the Institutional Animal Care and Use Committee of National Yang-Ming University (IACUC No. 1070311). All behavioral tests were performed during the light phase.

### 2.3. Pentobarbital-Induced Sleep Test

Mice were orally administrated 0.2 mL PBS or *L. fermentum* suspensions via an orogastric tube daily for different tested days. The pentobarbital-induced sleep mouse experiment was performed as previously described [19]. Briefly, mice were intraperitoneally injected with pentobarbital sodium (50 mg/kg, Sigma, Saint Louis, MO, USA). When the mice lost their righting reflex after about 30 s, they were considered to be asleep. The period of time during which mice did not show stereotactic reflection was measured as sleep latency, and the period of time from falling asleep to exhibiting stereotactic reflection was measured as sleep duration. The period of time from exhibiting stereotactic reflection to the recovery of free movement was measured as recovery time [19,20,21]. Mice injected with pentobarbital that did not fall asleep within 15 min were excluded from the experiment. Sacrifice was performed 2 h after the end of the experiment, and the mouse brain was quickly removed and temporarily put on dry ice. After that, the brain area of interest was taken out and stored at −80 °C. The orally administrated diphenhydramine hydrochloride (DIPH, 20 mg/mL, Sigma), an antihistamine with sleep aid effects, was used in the drug control group. Caffeine (15 mg/mL, Sigma) was intraperitoneally injected 30 min before the pentobarbital-induced sleep test to induce sleep disturbance in mice [21,22].

### 2.4. Open Field Test

The locomotor activity of the mice was examined using the open field test as previously described [23]. In brief, each mouse was placed in an arena with Plexiglas walls (25.4 × 25.4 × 38 cm^3^) with photobeam sensors to record locomotor activities for 10 min (Tru Scan Activity System; Coulbourn Instruments, Whitehall, PA, USA). The center area was defined as a region (12.5 × 12.5 cm^2^) in the center of the arena. The box was cleaned with 70% ethanol after each test. Increasing time spent in the central square and the ratio of central to total locomotion are considered indications of anxiolysis [24].

### 2.5. Genomic DNA Extraction and PCR Analysis of L. fermentum Strains

*L. fermentum* strains were cultured in MRS broth overnight. Bacterial cells were collected by centrifugation at 12,000× *g* for 5 min. The genomic DNA was extracted as previously described [25]. The purity of genomic DNA was evaluated in terms of the A260/A280 ratio with a NanoDrop 1000 spectrophotometer (Thermo Fisher Scientific Inc., Waltham, MA, USA). To discriminate the three *L. fermentum* strains, four PCR-based genomic fingerprinting methods were used, which include BOX-, (GTG)_5_-, enterobacterial repetitive intergenic consensus (ERIC)-, and random amplification of polymorphic DNA (RAPD)-PCR (Table 1). The PCR products were analyzed by electrophoresis using 2% agarose gel with a 1× TBE (tris-borate-EDTA) buffer. The gel was stained with ethidium bromide and observed under an ultraviolet transilluminator. A 100 bp ladder (Omics Bio, New Taipei city, Taiwan) was included as a molecular weight marker.

### 2.6. Quantitative Real-Time Polymerase Chain Reaction (qRT-PCR) Analysis

The total RNA in the brain tissue was extracted by using an RNeasy mini kit (Qiagen, Germantown, MD, USA) [28] and converted into cDNA using a RevertAid First Strand cDNA Synthesis kit (ThermoFisher, Waltham, MA, USA). The cDNA samples in each group were diluted 20-fold with DNase-free water and were subjected to two independent repetitions of real-time PCR with specific primers (Table 1) and KAPA SYBR FAST ABI Prism (KAPA Biosystems, Woburn, MA, USA) using the StepOnePlus™ Real-Time PCR System (Applied Biosystems, Foster City, CA, USA). The cycling conditions of qRT-PCR were 95 °C for 3 min; up to 40 cycles of 95 °C for 3 s, 60 °C for 30 s; and the melt curve stage was 95 °C for 15 s, 60 °C for 30 s, and 95 °C for 15 s. The target threshold cycle (Ct) was subtracted from the Ct for glyceraldehyde-3-phosphate dehydrogenase (GAPDH) to calculate ΔCt, and a relative quantification analysis was performed via the 2^−ΔΔCT^ method [29].

### 2.7. Statistical Analysis

Data were analyzed using GraphPad Prism 5.1 (GraphPad Software) and represented as the mean ± standard error of the mean (SEM). For multiple comparisons, we used a one-way analysis of variance (ANOVA) with Tukey’s post hoc test. A *p*-value < 0.05 was considered significant in all cases.

## 3. Results

### 3.1. Strain-Specific Effects of PS150^TM^ on Pentobarbital-Induced Sleep in Mice

**Figure 1:**
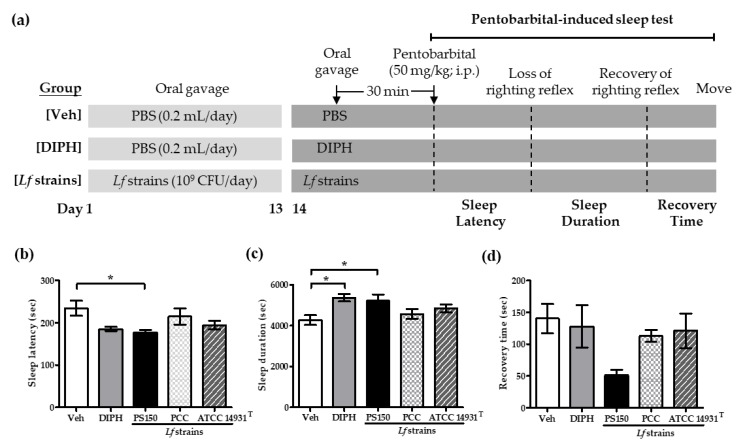
Effects of the oral administration of *L. fermentum* (*Lf*) strains on pentobarbital-induced sleep in mice. (**a**) The experimental design; mice were orally administrated phosphate-buffered saline (PBS) or 10^9^ colony-forming units (CFU) of *Lf* strains, including PS150^TM^, PCC, and ATCC 14931^T^, for 14 consecutive days. On experimental day 14, a pentobarbital (50 mg/kg)-induced sleep test was performed, and the sleep latency (n = 10~12) (**b**), sleep duration (n = 10~12) (**c**), and recovery time (n = 4~5) (**d**) of mice were recorded. Diphenhydramine (DIPH) (20 mg/kg) was introduced 30 min before the test. Veh refers to the vehicle control group. Data are expressed as mean ± standard error of mean (SEM) and were analyzed by one-way ANOVA with Tukey’s post hoc test. * *p* < 0.05, compared with the indicated groups.

The health benefits of probiotics are generally considered to be strain-specific. To examine if PS150^TM^ harbors a sleep-improving effect with strain-specificity, a pentobarbital-induced sleep mouse model was used, and the activity of different *L. fermentum* strains was evaluated (Figure 1a). Compared with the vehicle control (Veh) group, the oral administration of PS150^TM^ for 14 consecutive days significantly decreased the sleep latency (n = 10~12) and increased the sleep duration (n = 10~12) (Figure 1b,c); the recovery time (n = 4~5) was also decreased, but not significantly (Figure 1d). However, the other two *L. fermentum* strains, PCC (a commercial probiotic for gastrointestinal health) and ATCC 14931^T^ (the taxonomic type strain of *L. fermentum*), did not show any sleep-improving effects. Moreover, the oral administration of a well-known antihistamine drug used as a sleep aid, diphenhydramine (DIPH), only showed increased sleep duration, but no improvement in sleep latency or recovery time was observed on day 14 of our experiment, which is consistent with previous findings [30,31].

**Figure 2:**
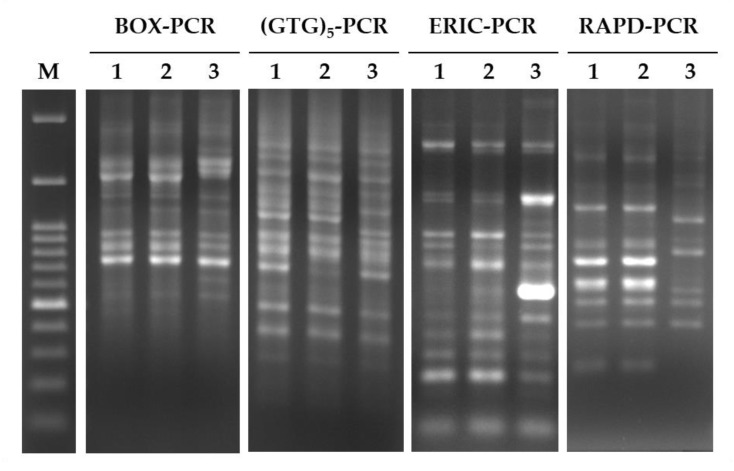
PCR-based genomic fingerprinting profiles of *L. fermentum* strains. Enterobacterial repetitive intergenic consensus (ERIC)-, BOX-, (GTG)_5_-, and random amplification of polymorphic DNA (RAPD)-PCR analyses were performed to discriminate *L. fermentum* strains. Lanes: 1, PS150^TM^; 2, PCC; 3, ATCC 14931^T^. M denotes the 100 bp ladder (Omics Bio).

To determine whether the *L. fermentum* strains under investigation have similar genetic backgrounds, three types of rep-PCR (ERIC, BOX, and (GTG)_5_) and an RAPD-PCR were performed, and the PCR products were resolved by DNA electrophoresis. As shown in Figure 2, PS150^TM^ and PCC appeared to have a similar genetic background, while we observed distinguishable PCR-fingerprinting profiles between the two strains and ATCC 14931^T^, especially in the ERIC-PCR profiles, suggesting two lineages of these *L. fermentum* strains. It is noteworthy that although PS150^TM^ and PCC appeared to be classified into the same lineage, only PS150^TM^ showed sleep-improving effects in our experiment.

### 3.2. PS150^TM^ Improved Pentobarbital-Induced Sleep in Mice in a Time- and Dose-Dependent Manner

**Figure 3:**
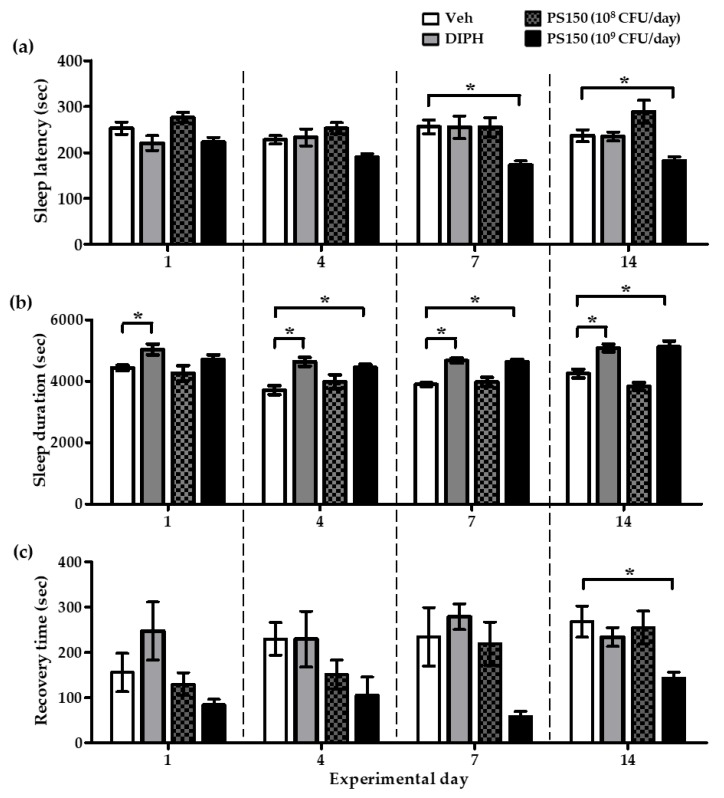
Effects of oral administration of *L. fermentum* PS150^TM^ with different intervention times and doses on pentobarbital-induced sleep in mice. Mice were orally administrated with PBS, 10^8^, or 10^9^ CFU of PS150^TM^ for 14 consecutive days. Pentobarbital (50 mg/kg)-induced sleep tests were performed on experimental days 1, 4, 7, and 14, and the sleep latency (n = 8~10) (**a**), sleep duration (n = 8~10) (**b**), and recovery time (n = 5~7) (**c**) of mice were recorded. DIPH (20 mg/kg) was introduced 30 min before the test. Veh refers to the vehicle control group. Data are expressed as mean ± SEM (n = 5~10) and were analyzed by one-way ANOVA with Tukey’s post hoc test. * *p* <0.05, compared with the indicated groups.

To investigate the effects of PS150^TM^ supplementation with different intervention times and daily dosages on sleep improvement, mice were orally administrated 10^8^ or 10^9^ CFU/day of PS150^TM^ for 14 consecutive days, and the pentobarbital-induced sleep tests were performed on days 1, 4, 7, and 14 (Figure 3). Compared with the vehicle control (Veh) group, the oral administration of PS150^TM^ of 10^9^ CFU/day significantly decreased the sleep latency (n = 8~10) on days 7 and 14, increased the sleep duration (n = 8~10) on days 4, 7, and 14, and decreased the recovery time (n = 5~7) on day 14, suggesting that the intervention time was critical for the sleep-improving effects to occur. However, treatment with a lower dosage (10^8^ CFU/day) did not affect the sleep pattern of mice. The oral gavage of DIPH to the drug control group 30 min before the pentobarbital-induced sleep test resulted in significantly increased sleep duration on all tested days compared with the Veh group (Figure 3b). Nevertheless, no significant difference in sleep latency or recovery time between the DIPH group and the Veh group was found (Figure 3a,c).

### 3.3. Effects of PS150^TM^ on Caffeine-Induced Sleep Disruption in Mice

**Figure 4:**
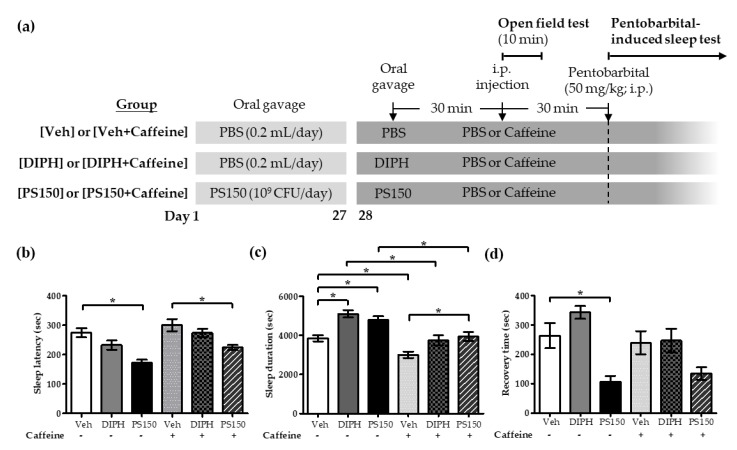
Effects of oral administration of *L. fermentum* PS150^TM^ on caffeine-induced sleep disturbance in mice. (**a**) Experimental design; mice were orally administrated PBS or 10^9^ CFU of PS150^TM^ for 28 consecutive days and subjected to an injection of PBS or caffeine (15 mg/kg), and a pentobarbital (50 mg/kg)-induced sleep test was performed to evaluate the sleep latency (n = 13~15) (**b**), sleep duration (n = 13~15) (**c**), and recovery time (n = 4~6) (**d**) of mice. DIPH (20 mg/kg) was introduced 30 min before the test. Veh refers to the vehicle control group. Data are expressed as mean ± SEM and were analyzed by one-way ANOVA with Tukey’s post hoc test. * *p* <0.05, compared with the indicated groups.

To further investigate the potential of PS150^TM^ as a sleep-improving dietary supplement, the effects of PS150^TM^ on caffeine-induced sleep disturbance in mice were evaluated. Mice were orally administrated PBS or PS150^TM^ (10^9^ CFU/day) for 28 consecutive days and then intraperitoneally injected with PBS or caffeine (15 mg/kg), subjected to an open field test for 10 min, and then subjected to a pentobarbital-induced sleep test. The oral gavage of DIPH (20 mg/kg) was used as a drug control group (Figure 4a). As shown in Figure 4b, the oral administration of PS150^TM^ to mice, either with or without the caffeine injection, significantly decreased the sleep latency (n = 13~15) (*p* < 0.05), while no significant effect of caffeine and DIPH was observed. Furthermore, compared with the Veh group, both the DIPH and the PS150^TM^ groups showed increased sleep duration (n = 13~15) (Figure 4c); the caffeine injection decreased the sleep duration, which could be attenuated by the supplementation of PS150^TM^, but not DIPH, implying the stronger sleep-improving effects of PS150^TM^. Furthermore, the supplementation of PS150^TM^ significantly decreased the recovery time only in mice without caffeine injection (*p* <0.05), and no other statistically significant differences were found (n = 4~6) (Figure 4d). These results indicate that the daily supplementation of PS150^TM^ for 28 days attenuated the caffeine-induced sleep disturbance in mice. Nevertheless, this sleep-improving effect of PS150^TM^ could not be observed on experimental day 14 (data not shown), supporting the notion that the sleep-improving effects of PS150^TM^ are time-dependent.

### 3.4. PS150^TM^ Did Not Alter the Spontaneous Locomotor Activity of Mice in the Open Field Test

**Figure 5:**
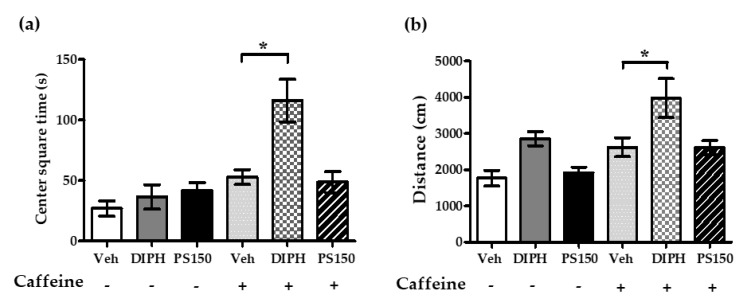
The open field test indicated that the oral administration of DIPH, but not PS150^TM^, affected locomotor activity in mice: (**a**) center square time, (**b**) total distance. The experimental design is shown in Figure 4a; a 10-min open field test was performed after the injection of PBS or caffeine (15 mg/kg). PS150^TM^ was orally administrated for 28 consecutive days. DIPH (20 mg/kg), as a drug control group, was introduced on experimental day 28. Data are expressed as mean ± SEM (n = 8~10) and were analyzed by one-way ANOVA with Tukey’s post hoc test. * *p* < 0.05, compared with the indicated groups.

As shown in Figure 4a, a 10-min open field test was performed to analyze the effects of PS150^TM^ on the locomotor activity of mice with or without the intraperitoneal injection of caffeine (n = 8~10). The drug control group of DIPH, orally administrated only on experimental day 28, was also included in the test. The open field test indicated that, compared with the (Veh + Caffeine) group, the (DIPH + Caffeine) group showed increased time spent in the central square (Figure 5a) and increased total distance (Figure 5b), while no other significant differences were observed. PS150^TM^ did not alter the locomotor activity of the mice.

### 3.5. Effects of PS150^TM^ on the Expression of Genes Related to Adenosine Signaling in the Basal Forebrain and Hypothalamus

The inhibitory neurotransmitter adenosine is involved in sleep regulation, and the elevation of extracellular adenosine appears to be a crucial mechanism to increase sleep duration and electroencephalography (EEG) slow-wave activity [32,33]. The concentration of adenosine changes during spontaneous sleep in some sleep-related brain regions, including the basal forebrain, the preoptic area of the hypothalamus, etc. [33,34]. To explore whether PS150^TM^ alters sleep patterns by affecting the sleep-related pathways of mice undergoing the pentobarbital-induced sleep test, we used qRT-PCR to analyze the expression of genes encoding adenosine receptors A_1_ (A_1_R) and A_2A_ (A_2A_R) as well as NT5e, the nucleotidase that synthesizes adenosine (Figure 4a). As shown in Table 2, compared with the Veh group, the oral administration of PS150^TM^ for 28 consecutive days significantly increased the expression of A_1_R in the hypothalamus (*p* < 0.05) of the mice that were not subjected to the caffeine treatment. However, no other significant differences were observed in our experiment.

## 4. Discussion

Recent studies have correlated the gut microbiome with immune function, nutrient metabolism, circadian rhythms, and mood disorders [35,36]. Through the MGBA, the gut microbiome influences not only the digestive, immune, and metabolic functions but also the sleep and mental states of the host [37]. To manipulate the gut microbiome for improving host health, probiotics are generally utilized, mostly for digestive and immune functions [38,39]. There is considerable evidence showing that probiotics may be effective in reducing stress and anxiety and alleviating low moods [40]. However, specific probiotic strains that can be used for sleep improvement are rare. Dietary supplementation of heat-killed *Lactobacillus brevis* SBC8803 can modulate circadian locomotion and sleep rhythms, as assessed by an EEG analysis [41]. The oral administration of Japanese sake yeast promotes non-rapid eye movement (NREM) sleep in mice via the activation of adenosine A_2A_ but not A_1_ receptors [42]. Moreover, the daily consumption of *Lactobacillus casei* Shirota or heat-inactivated *Lactobacillus gasseri* CP2305 improved stress-related symptoms and sleep quality in exploratory clinical trials [43,44].

In this study, we used a pentobarbital-induced sleep mouse model to identify potential probiotics with sleep-improving effects, which is the most commonly used method for screening of sedative-hypnotic agents [45,46]. Numerous strains belonging to *L. fermentum* and other *Lactobacillus* spp. were tested, but only PS150^TM^ showed sleep-improving effects in our experiment. Pentobarbital is a barbituric acid that activates GABA type A receptors, leads to cellular hyperpolarization within the CNS, and produces dose-dependent sedation and hypnosis [47]. Sleep latency and sleep duration obtained in the pentobarbital-induced sleep test are commonly used as indicators for assessing the sedative and hypnotic effects of dietary supplements (most of them are plant extracts) and drugs including DIPH, diazepam (a longer-acting benzodiazepine), and 5-ydroxytryptophan (a clinically effective serotonin precursor) [21,46,48,49]. The administration of caffeine has also been shown to increase sleep latency and decrease sleep duration in a dose-dependent manner assessed by the pentobarbital-induced sleep test [50]. Moreover, the hypnotic effect and the interaction of caffeine with pentobarbital have been studied in 42 medical and surgical patients [51]. To our knowledge, this is the first study that used the pentobarbital-induced sleep mouse model to evaluate the potential sleep-improving effects of probiotics; moreover, the caffeine-induced sleep disturbance appeared to be ameliorated by the supplementation of PS150^TM^. However, these results are preliminary and need to be confirmed by further animal and clinical studies.

The dietary supplementation of PS150^TM^ for more than 14 consecutive days appeared to decrease the sleep latency and increase the sleep duration and recovery time of mice with pentobarbital-induced sleep. Sleep recovery, measured from the recovery of righting reflex to the beginning of a movement, reflects the subsequent effects of hypnotics on the CNS [21]. An ideal hypnotic should not affect awakening after sleep, and PS150^TM^ seemed to have this feature. As shown in Figure 3, on experimental day 1, the oral administration of PS150^TM^ did not affect the sleep pattern of mice. However, PS150^TM^ increased the sleep duration from day 4, decreased the sleep latency from day 7, and decreased the recovery time on day 14. We speculated that PS150^TM^ may need to colonize the gut mucosa and interact with the host microbiota to gradually regulate the sleep pattern via the MGBA. It is known that probiotics can alter the gut microbiota by competing for nutrients, producing antimicrobial compounds, or modulating host immunity [52]. The possibility that PS150^TM^ modulates the host gut microbiota remains to be investigated. On the other hand, the oral gavage of DIPH, which exerts its hypnotic effects via the antihistamine pathway [30], only prolonged sleep duration but did not affect sleep latency or recovery time. Based on these findings, we suggest that PS150^TM^ regulates sleep patterns differently from DIPH, although the exact mechanism is not yet clear.

Various neurotransmitters, including gamma-aminobutyric acid (GABA), serotonin or 5-hydroxytryptamine (5-HT), adenosine, histamine, and orexin, affect different brain nuclei to regulate the switch between wakefulness and sleep [2,53]. Although PS150^TM^ has been demonstrated to prevent the stress-mediated reduction of 5-HT and neurodegeneration in the rat brain, in this study, the oral administration of PS150^TM^ did not affect the levels of 5-HT or 5-hydroxyindole acetic acid (5-HIAA) in specific mouse brain areas (striatum, prefrontal cortex, hippocampus, and hypothalamus) assessed by high-performance liquid chromatography-electrochemical detection (data not shown). Specific strains of *Lactobacillus* spp. can produce GABA, the chief inhibitory neurotransmitter in the mammalian CNS. The ability to synthesize GABA is considered to be an important feature of psychobiotics [54]. However, PS150^TM^ did not seem to produce GABA in vitro from its precursor monosodium glutamate, as assessed by a thin-layer chromatography analysis of its culture supernatant (data not shown). However, PS150^TM^ appeared to increase the expression of adenosine A_1_R in the hypothalamus (Table 2). Adenosine is an inhibitory neurotransmitter that has been proposed to decrease the activity of orexinergic and histaminergic neurons via A_1_R in different areas of the hypothalamus to promote sleep [55,56,57]. Moreover, the histaminergic output from the hypothalamus plays an important role in mediating forebrain arousal [58]. Whether or not the sleep-improving effects of PS150^TM^ are involved in these adenosine A_1_R-mediated regulations in the hypothalamus awaits further investigation.

Although its pathogenesis is not fully understood, important features of insomnia include difficulty initiating or maintaining sleep, and waking up earlier than desired [59]. Many studies have shown that insomnia is often accompanied by neurophysiological abnormalities and poor health [5]. The use of medications is a dominant approach to treat insomnia, which involves four fundamental pharmacodynamic categories with key actions related to receptors of GABA, melatonin, histamine, or orexin/hypocretin [59]. Nevertheless, side effects associated with hypnotics are common [59]. Thus, the development of alternative treatment strategies with higher safety, including dietary supplements, is needed. Probiotics, which include strains of *Lactobacillus*, *Bifidobacterium*, and *Saccharomyces*, have a long history of safe and effective use as dietary supplements. Moreover, emerging evidence has shown the potential of psychobiotics to improve CNS-related illnesses, particularly in stress-related, anxiety, and depressive disorders [60]. In this study, we have shown the potential sleep-improving effects of PS150^TM^, which has also been demonstrated as a psychobiotic capable of alleviating abnormal behaviors induced by mild chronic stress in rats [16]. The use of PS150^TM^ as a dietary supplement is generally considered as safe, since PS150^TM^ is classified as the species *L. fermentum,* with the qualified presumption of safety (QPS) status suggested by the European Food Safety Authority [61]. One major limitation of this study is the non-use of polysomnography; moreover, possible interactions between pentobarbital and probiotics cannot be excluded. To further understand the sleep-improving effects of PS150^TM^, we are now performing EEG and electromyogram analyses to investigate if PS150^TM^ affects sleep architecture in mice.

## 5. Conclusions

The present data demonstrate that the psychobiotic strain *L. fermentum* PS150^TM^ can potentially bring sleep-improving effects to both normal and caffeine-treated (mimicking short-term insomnia) mice without affecting their locomotor activities. The potential hypotonic effects of PS150^TM^ are strain-specific, showing some time- and dose-dependency, and may be involved in the regulation of the histaminergic system via adenosine A_1_ receptor. This suggests a new direction for the future development of novel dietary supplements as sleep aids.

## Figures and Tables

**Table 1:** Primers used in the present study.

Primer	Sequence (5′→3′)		Reference
ERIC	ATGTAAGCTCCTGGGGATTCAC		[26]
	AAGTAAGTGACTGGGGTGAGCG		
BOX	CTACGGCAAGGCGACGCTGACG		[16]
(GTG)_5_	GTGGTGGTGGTGGTG		[27]
RAPD	CTCAGGTCGC		Present work
**For qRT-PCR**	**Sequence (5′→3′)**	**Size (bp)**	**Accession number**
A_1_R-F	AGAACCACCTCCACCCTTCT	227	XM_006529079.2
A_1_R-R	TACTCTGGGTGGTGGTCACA		
A_2A_R-F	AACCTGCAGAACGTCAC	245	XM_006513093.3
A_2A_R-R	GTCACCAAGCCATTGTACCG		
NT5e-F	TTACTAAAGCATGACTCTGGTGATCAA	84	NM_011851.4
NT5e-R	AACGGCTGGGTAAACTACTTTCATT		
GAPDH-F	CAATGTGTCCGTCGTGGATCT	208	XM_017321385.1
GAPDH-R	GTCCTCAGTGTAGCCCAAGATG		

ERIC, enterobacterial repetitive intergenic consensus; RAPD, random amplification of polymorphic DNA; qRT-PCR, quantitative real-time polymerase chain reaction; A_1_R, adenosine A_1_ receptor; A_2A_R, adenosine A_2A_ receptor; NT5e, 5’ nucleotidase-ecto—enzyme that converts adenosine monophosphate to adenosine; GAPDH, glyceraldehyde 3-phosphate dehydrogenase.

**Table 2:** Quantitative Real-Time Polymerase Chain Reaction (qRT-PCR) analysis of the expression of genes encoding adenosine receptors and nucleotidase in the basal forebrain and hypothalamus of mice.

Gene Name	Basal Forebrain				Hypothalamus			
	Veh	PS150	Veh + Caffeine	PS150 + Caffeine	Veh	PS150	Veh + Caffeine	PS150 + Caffeine
A_1_R	1.00 ± 0.13	0.91 ± 0.06	0.86 ± 0.17	1.00 ± 0.15	1.00 ± 0.11	1.38 ± 0.19 *	1.08 ± 0.18	1.03 ± 0.11
A_2A_R	1.00 ± 0.21	0.90 ± 0.46	1.50 ± 0.3	1.39 ± 0.28	1.00 ± 0.14	0.95 ± 0.23	0.88 ± 0.15	0.89 ± 0.22
NT5e	1.00 ± 0.15	0.83 ± 0.19	0.90 ± 0.15	0.97 ± 0.22	1.00 ± 0.06	1.02 ± 0.23	1.09 ± 0.17	1.12 ± 0.23

The experimental design is shown in Figure 4a; the expression of genes was analyzed in the basal forebrain (n = 4~5) and hypothalamus (n = 6~8) of mice. Data are expressed as mean ± SEM and were analyzed by one-way ANOVA with Tukey’s post hoc test. * *p* <0.05, compared with the Veh group.
